# Heritability for body colour and its genetic association with morphometric traits in Banana shrimp (*Fenneropenaeus merguiensis*)

**DOI:** 10.1186/s12863-014-0132-5

**Published:** 2014-12-05

**Authors:** Nguyen Hong Nguyen, Jane Quinn, Daniel Powell, Abigail Elizur, Ngo Phu Thoa, Josephine Nocillado, Robert Lamont, Courtney Remilton, Wayne Knibb

**Affiliations:** University of the Sunshine Coast, Locked Bag 4, Maroochydore DC, Queensland 4558 Australia; Seafarm, Bruce Hwy, Cardwell, QLD 4849 Australia

**Keywords:** Genetic improvement, Selection, Meat quality and shrimp breeding

## Abstract

**Background:**

Banana shrimp *Fenneropenaeus merguiensis* has emerged as an important aquacultured shrimp species in South East Asia and Australia. However, the quantitative genetic basis of economically important traits in this species are currently not available, while for body colour, cooked or uncooked, there are no genetic parameter estimates for any shrimp or indeed any decapod crustacean. In this study, we report for banana shrimp genetic parameters for morphometric traits and, the first time for any shrimp, parameter estimates for body colour. Ten highly polymorphic microsatellite markers were developed from genomic sequences and used to construct a pedigree for 2000 offspring from approximately 60 female and 60 male parents that were sampled from a single routine commercial production pond.

**Results:**

Restricted maximum likelihood method applied to a single trait mixed model was used to estimate heritabilities, while correlations were estimated using the multi-trait approach. The estimates of heritability for morphometric traits were moderate to high (h^2^ = 0.14 – 0.50). Body colour of uncooked shrimp showed a heritable additive genetic component (h^2^ = 0.03 – 0.55), and those estimates obtained for cooked shrimp were significantly different from zero. Genetic correlations among morphometric traits were all positive and very high (close to unity, r_g_ = 0.85 – 0.99). The genetic correlations of body traits (weight, length and width) were positive with both colour after cooking (0.74 – 0.84) and body colour measured on live shrimp (0.59 to 0.70). The positive genetic correlations between the cooked body colour and uncooked body colour (0.64 ± 0.20) suggests these two traits can be simultaneously improved in practical selective breeding programs. This first ever report of genetic parameters for cooked or uncooked colour in crustacean indicates there is potential for genetic improvement of both growth and body colour through selection.

**Conclusions:**

In the present study we demonstrated for banana shrimp that genetic parameters can be estimated from commercial samples (using pedigrees based on DNA markers), that selection for shrimp colour should be successful under such commercial conditions.

**Electronic supplementary material:**

The online version of this article (doi:10.1186/s12863-014-0132-5) contains supplementary material, which is available to authorized users.

## Background

Banana shrimp *Fenneropenaeus merguiensis* accounts for about 30% of Australian shrimp aquaculture production (i.e. about 1,300 tons), while there is significant production in Asian countries such as Indonesia and Vietnam [1]. Banana shrimp are readily bred in captivity without artificial insemination (AI) and so are agreeable candidates for selection. This contrasts the situation for black tiger shrimp (*Penaeus monodon*), currently the dominant aquacultured shrimp species in Australia, where breeding pond reared animals is problematic and AI is required to construct even limited pedigrees [2].

Banana shrimp, along with *Litopenaeus vannamei*, are considered ‘white shrimp’ that are generally more tender and preferred by some food sectors to counterpart species. Increasing the redness of white shrimp is seen as desirable by some industry sectors and promote premium pricing. For example in Australia, highest scoring coloured shrimp assessed using subjective colour scoring system are often priced AU$2-4/ kg more than light-coloured shrimp [3]. Shrimp colour is largely dependent on the amount of astaxanthin present in external tissues (the exoskeleton and the epidermal layer) [4]. A common practice to improve shrimp colour is through supplement of synthetic astaxanthin in the diets [5]. Several other rearing and harvesting factors (particularly pre- and post-slaughter conditions) such as transportation, colour of holding containers, handling, conditioning, fasting, killing method, chilling and storage may have influence on shrimp colour [6]. In contrast to the abundant evidence that shrimp colour can be improved through manipulation of environmental factors and husbandry practices, there has been a paucity of scientific research in quantitative genetic aspects of shrimp colour. Genetic variation in body colour within shrimp species, strains or lines is not known, and genetic relationships of body colour with morphometric traits have not been estimated in all crustaceans. Indeed, it is unknown whether there is genetic variance in banana shrimp for colour or redness, and so whether it would be possible to select on this trait. Moreover, it is unknown whether selection for colour would have adverse effects on other commercial traits such as body weight and length.

Therefore, the principal aim of our present study was to examine the quantitative genetic basis of body colour and its genetic associations with morphometric traits in Banana shrimp *F. merguiensis.* To support this study, ten highly polymorphic microsatellite markers were developed for banana shrimp and they were used to construct the pedigree for quantitative genetic analysis.

## Methods

### Experimental location

The study was approved by the animal ethics committee of the University of the Sunshine Coast. The experimental field work was conducted at Seafarm, in Cardwell, North Queensland (latitude 18° 16′ 0S, longitude 146° 1′ 60E, altitude 0 m). The daily average temperature in Cardwell is between 14 and 32°C, with the minimum average of 19°C and the maximum average of 29°C over the last 103 years (Australian Bureau of Meteorology) [7]. The water temperature in cultured ponds varies between 25°C and 32°C. The annual rainfall is 2129 mm, occurring mainly from December to April with a peak in January, February and March.

### Origin of the animals

The animals originated from a population mass selected for length over 14 generations, which more recently had gone through several rounds of intercrossing among the long term lines. A detailed description of the population is given in Knibb et al. [8]. In brief, six lineages (cohorts) were formed in 2000 from twenty wild inseminated females and they were bred in captivity for 14 or more generations. The typical breeding cycle is given in Knibb et al. [8]. Grow-out followed a standard commercial practice. The shrimp were fed an amount equivalent to 3 to 5% of their live weight on a commercial dry pelleted feed with 38% protein content four times a day (i.e. at 6:00a.m, 10:00 a.m., 3:00 p.m. and 6:00 p.m.). The inital stocking density in each pond was 50 shrimp per square meter of surface water. Water quality parameters (temperature, pH, dissolved oxygen and total ammonia) were also monitored once a week. After about 140 days of grow out, the shrimp were sampled in this study.

### Sample collection and measurements

Shrimp were sampled using a cast net (mesh size of 2 cm) in small batches averaging 80 animals from a grow-out pond at eight different locations around the pond (East, North, North Conner, North Walkway, South, South West, Southern End and West). The harvested shrimp were then transferred to aerated containers of uniform red colour (0.7 × 0.5 × 1.5 m) at a very low density (20 shrimp per container). Data recording was conducted within one to two minutes of placing the animals in the tubs. Body traits measured on each individual were live weight (total live body weight in gram), total body length (distance from rostrum to tip of telson in cm), head (cephalothorax) length (distance from eye orbit to the hind margin of the carapace, cm), abdominal length (distance from the hind margin of the carapace to tip of telson, cm) and abdominal width (width of the second abdominal segment, cm). Recording carcass weight trait included only tail (abdominal) weight (weight of the tail segment, g) and was used to calculate meat yield (expressed as percentage of tail weight to total live weight, %). In addition to body and carcass traits, sex, culture pond, sampling time and sampling location within the pond were also recorded at measurements. The visual assessment of body colour was scored subjectively as light or dark on individual raw (uncooked) shrimp. After recording the data, the shrimp tail segments were kept in separate plastic bags and labelled according to our tag system for individual identification. All shrimp were immediately stored in cold insulated ice chests (3°C) and then transferred to a cold room (−10°C) before cooking within six hours to measure cooked colour and ‘flesh streaks’ back. Shrimp were cooked in a commercial facility for three minutes following standard commercial practices. Cooked colour of individual shrimp was recorded as light or red. The trait of ‘flesh streaks’ on the back was described as mushy, soft and chalky texture of the cooked shrimp and was recorded as presence or absence on individual shrimp together with cooked colour. In addition, hepatopancreas samples were taken from individual shrimp following morphometric measurement. Hepatopancreas samples were preserved in RNA later and shipped to University of the Sunshine Coast.

### Genotyping and pedigree construction

Ten microsatellite loci (GenBank Accession No’s: KM213743-KM213752) with consistent PCR amplification, clear allelic variation, and clarity of electrophoretic signatures were used to construct the pedigree in the present study (Additional file 1). A detailed description of marker development from the pooled genomic DNA of 20 *F. merguiensis* individuals using GS-FLX Titanium chemistry (Roche Applied Science; Mannheim, Germany) is given in Knibb et al. [8,9].

**Figure 1:**
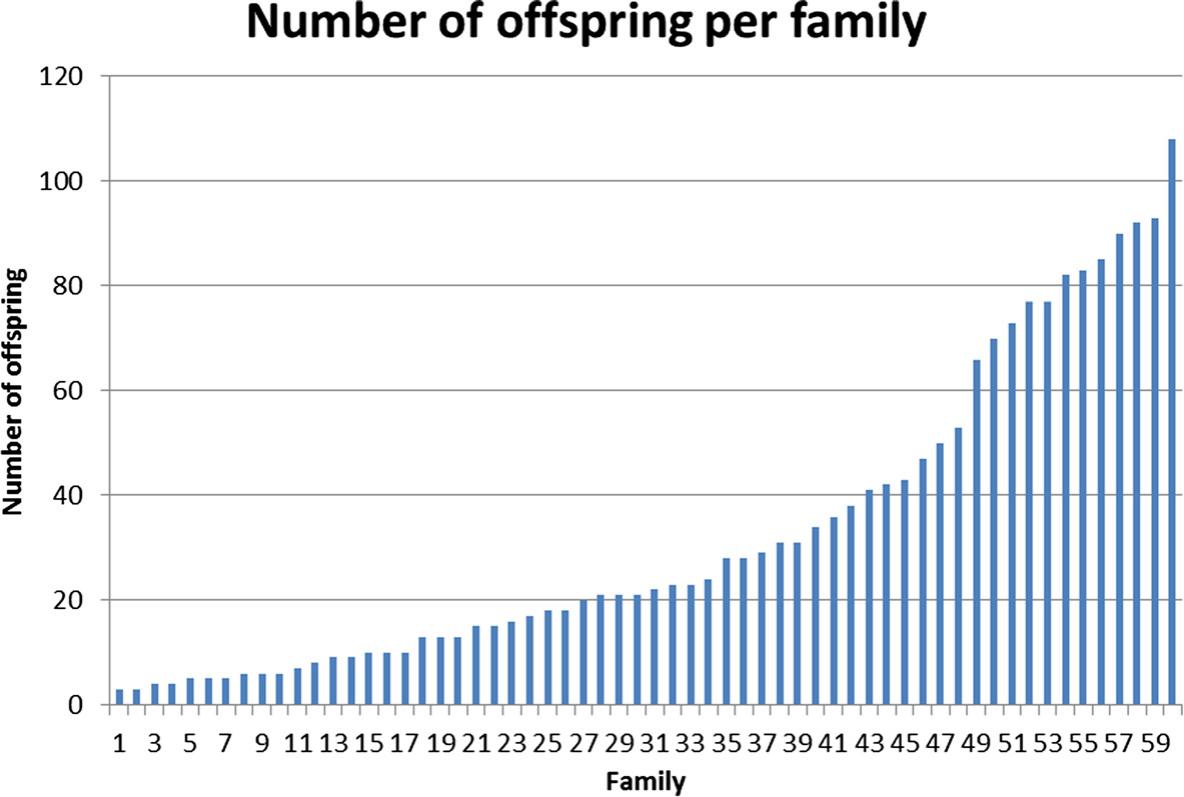
**Number of offspring per family assuming monogamy.**

Once validated in simplex, two multiplex PCR pools, each containing 5 microsatellite primer pairs (Pool 1: *FM002*, *FM004*, *FM011*, *FM047*, *FM057*, and Pool 2: *FM001*, *FM005*, *FM014*, *FM052*, *FM056*) were amplified using Qiagen Multiplex PCR Plus Kits (Qiagen, Germany). Forward and reverse primers for each multiplex pool were combined in a 10× primer mix using 1–3 μM of each primer, dependent upon PCR product fluorescence intensities. Reactions, with volumes adjusted to 10 μL, each contained 1 μL of 10× primer premix, 3.0 μL of Qiagen Multiplex Buffer (2×) buffer, 3.5 μL of DH_2_O, and 2.5 μL of template gDNA (10 ng/μL). Amplification was performed using an Eppendorf Mastercycler (Hamburg, Germany) with cycling conditions as follows: initial denaturation at 95°C for 5 min, followed by 35 cycles of 94°C for 30 s, 57°C for 90 s, and 72°C for 30 s; with a final extension at 68°C for 10 min. PCR products were separated by capillary electrophoresis on an AB 3500 Genetic Analyser (Applied Biosystems). Fragment sizes were determined relative to an internal lane standard (GS-600 LIZ; Applied Biosystems) using GENEMARKER v1.95 software (SoftGenetics; State College, USA) and double-checked manually. Individuals with low or missing peaks were amplified and genotyped a second time. MICRO-CHECKER v2.2.3 [10] was used to look for evidence of large allele dropout, extreme stuttering and null alleles, based on 1000 bootstraps and a 95% confidence interval. Tests for HWE at each locus and genotypic linkage equilibrium among pairs of loci were conducted in FSTAT v2.9.3 [11]. Numbers of alleles and the observed and expected heterozygosities of each locus were determined using GENALEX v6.5 [12], while polymorphic information content (*PIC*) was computed in CERVUS v3.0 [13]. Parentage assignment was completed using COLONY software [14] with confidence scores of above 95%. Our earlier study using both mtDNA and microsatellite markers [9] showed the evidence of monogamy in this banana prawn population. Thus the monogamy model was assumed to construct the pedigree that included 60 full-sib groups, with the family size of 3 to 108 offspring. A total of 1957 offspring out of 1998 were assigned to full sib families. This previous study [9] also reported pedigrees constructed using these microsatellite loci contained very few errors when cross checked with independent mtDNA sequence data. The number of offspring per family is given in Figure 1. The pedigree data file with phenotypes is available on request.

### Statistical analysis

#### Data and exploratory analysis

Exploratory analyses were firstly performed to detect possible errors and examine distribution of the data for all traits studied. The sample statistics (skewness and kurtosis values) for all body traits were close to zero indicating that the data were normally (or approximately normally) distributed. Transformations (e.g. square root or logarithm) did not improve the distribution of the data and hence all analyses for body traits were performed on original scale of measurements. Analysis of variance using linear fixed model was used to examine systematic factors to determine the final statistical models for each trait. All analyses were conducted in SAS 9.3 (SAS Inc) [15].

### Linear mixed model

Genetic parameters for all traits studied were analysed using linear general mixed model in ASReml [16]. The model included the effects of sampling time (AM, PM), operator (2 technicians), sex (female and male) and sampling batch by location within the pond subclass (Equation 1). The random term in the model was the additive genetic effect of individual shrimp in the pedigree. In the present study all families were pooled early (as soon as hatching) and then raised communally; thus the effect common to full-sib groups (c^2^) was not included in the final model. The logarithmic likelihood ratio test showed that the c^2^ effect of dam (a combination of maternal, environmental and partially dominant effects) was not significant for all body traits (Chi-square test with one degree of freedom, *P* > 0.05). This is consistent with our observations in yellowtail kingfish [17] and also in other studies [18,19]. In a mathematical form, the model is written as the following:

1 $$ {y}_{ijklmn}=\mu + B{L}_i + {O}_j + {S}_k + {T}_l+{\beta}_m\left({W}_m\right) + {a}_n + {e}_{ijklmn} $$

where *y*_*ijkl*_ is the observation of an individual (traits studied), *μ* is mean and the effects of sampling batch and location subclass (BL)_*i*_ (*i* = 1 to 25), operators O_*j*_ (*j* = 2), sex S_*k*_ (*k* = 2, female and male) and sampling time T_*l*_ (*l* = 2, AM and PM), and *β*_*m*_(*W*_*m*_) is a linear regression coefficient of weight fitted for body colour. The additive genetic effect (*a*) is assumed *a*$\sim \left(0,\mathrm{A}{\sigma}_a^2\right)$ where **A,** is the additive genetic (numerator) relationship matrix among the animals that was calculated directly from the pedigree, and ***e*** is the vector of residual effects $\sim \left(0,\mathrm{I}{\sigma}_e^2\right)$.

Under linear mixed model (Eq. 1), heritabilities for morphometric traits and body colour were estimated from a single trait model. Phenotypic and genetic correlations were obtained from a series of bivariate analyses, using the same statistical model as described above. Heritabilities for body traits and colour were calculated as ${h}^2=\frac{{\widehat{\sigma}}_a^2}{{\widehat{\sigma}}_a^2+{\widehat{\sigma}}_e^2}$ where ${\sigma}_a^2$ is the additive genetic variance and $\left({\sigma}_e^2\right)$ is the residual variance. Genetic and phenotypic correlations among traits were calculated as the covariance divided by the product of the standard deviations of traits: $r=\frac{\sigma_{XY}}{\sqrt{\sigma_X^2}\sqrt{\sigma_Y^2}}$ where *σ*_*XY*_ was the estimated additive genetic or phenotypic covariance between the two traits, and ${\sigma}_X^2$ and ${\sigma}_Y^2$ are the additive genetic or phenotypic variances of traits X and Y, respectively.

### Threshold generalised linear mixed model (GLMM)

In addition to linear mixed model, body colour of raw shrimp were measured in the form of ‘light’ or ‘dark as binary traits (coded as 0 and 1) and were also analysed using different threshold models with both logit and probit link functions. Similarly for cooked shrimp, body colour was measured as ‘light red’ or ‘dark red’. The former model assumed that the data followed a binomial distribution with a logit link functions ($\widehat{p}$ = e^x^ /(1 + e^x^)) where p is the probability of dark (or red) colour recorded at harvest and *x* is a linear predictor. The model fitted was the same as equation 1, except operator for cooked colour because only one technician recorded this trait. Means of body colours were back-transformed from the logit scale to the proportional observations. With GLMM sire model, heritability was calculated using the variance of the logit link function, which implies a correction of the residual variance by factor π^2^/3.

$$ {h}^2=\frac{4{\sigma}_s^2}{\sigma_s^2+{\sigma}_e^2\frac{\pi^2}{3}} $$

where ${\sigma}_s^2$ is sire variance and ${\sigma}_e^2=1.$

Probit threshold model.

The threshold sire model is basically the same as those described above. However, the probit link function *η* = *Φ*^− 1^(*p*_*i*_) is used, with inverse link ${p}_i=\varPhi \left(\eta \right)={\displaystyle \underset{-\infty }{\overset{\eta }{\int }}\frac{1}{\sqrt{2\pi }}{e}^{\frac{-{x}^2}{2}}dx}$, where *Φ* is the cumulative normal density function, and *p*_*i*_ denotes the probability of dark (or red) colour for shrimp *i*. The Bernoulli distribution for a binary trait for an individual shrimp with y_i_ = 1 (presence of dark colour in raw shrimp and of red colour in cooked shrimp) and y_i_ = 0 (absence) is the probability (y_i_|p_i_) = (p_i_)y_i_(1-p_i_)^1-y^_i_.

Under probit threshold model, heritability was calculated as

$$ {h}^2=\frac{4{\sigma}_s^2}{\sigma_s^2+{\sigma}_e^2} $$

where ${\sigma}_s^2$ is sire variance and ${\sigma}_e^2=1.$

For binomial observations, estimates of *h*^*2*^ on the liability scales (logit and probit) can be transformed to observed (0/1) scale using the formula of Robertson and Lerner [20] as follows:

$$ {h}_O^2={h}_L^2\frac{z^2}{p\left(1-p\right)} $$

where ${h}_O^2$ is the heritability on the observed (0/1) scale, ${h}_L^2$ is the estimated heritability on the liability (logit or probit) scale, *p* is a proportion of a given colour in the data, and *z* is the height of the ordinate of normal distribution corresponding to a truncation point applied to *p* proportion of colour.

The same methodology as described above was applied to estimate heritability for other binary traits (i.e. ‘flesh streaks’ and yellow hepatopancreas). Significance of the heritability estimates was tested using z-score against a large random normal distribution (e.g. Nguyen et al.) [21].

## Results

### Descriptors of the data

The unadjusted means, standard deviations and coefficients of variation for body colour and morphometric traits are shown in Table 1. The average body weight of the shrimp at harvest was about 17 g, corresponding to a tail weight of 10 g and edible meat yield of 60.7%. The proportion of cooked shrimp showing red colour was markedly lower compared to the dark colour recorded in raw shrimp (16.3 vs. 51.5%). The incidence of ‘flesh streaks’ was 16.9%, whereas it was only 1.65% for yellow hepatopancreas.

**Table 1:** **Number of data records (N), mean, standard deviation (SD), minimum and maximum values for traits studied**

**Traits**	**Unit**	**N**	**Mean**	**SD**	**Min**	**Max**
Body weight	g	1998	16.5	4.8	4.5	36.2
Body length	cm	1998	13.9	1.4	9.2	16.0
Head length	cm	1998	4.9	0.6	2.5	6.3
Body width	mm	1979	12.7	1.6	4.0	17.4
Tail weight	g	1997	10.0	10.0	2.9	2.5
Meat Yield	%	1989	60.7	3.8	47.1	75.3
Dark colour (raw shrimp)	%	1988	51.5	49.9	0	100
Red colour (cooked shrimp)	%	1998	16.3	36.9	0	100
‘Flesh streaks’	%	1998	16.9	37.5	0	100
Yellow hepatopancreas	g	1998	1.65	12.8	0	100

Basic statistics were estimated from about 2000 animals.

### Sampling location, time, operator and sex effect

**Figure 2:**
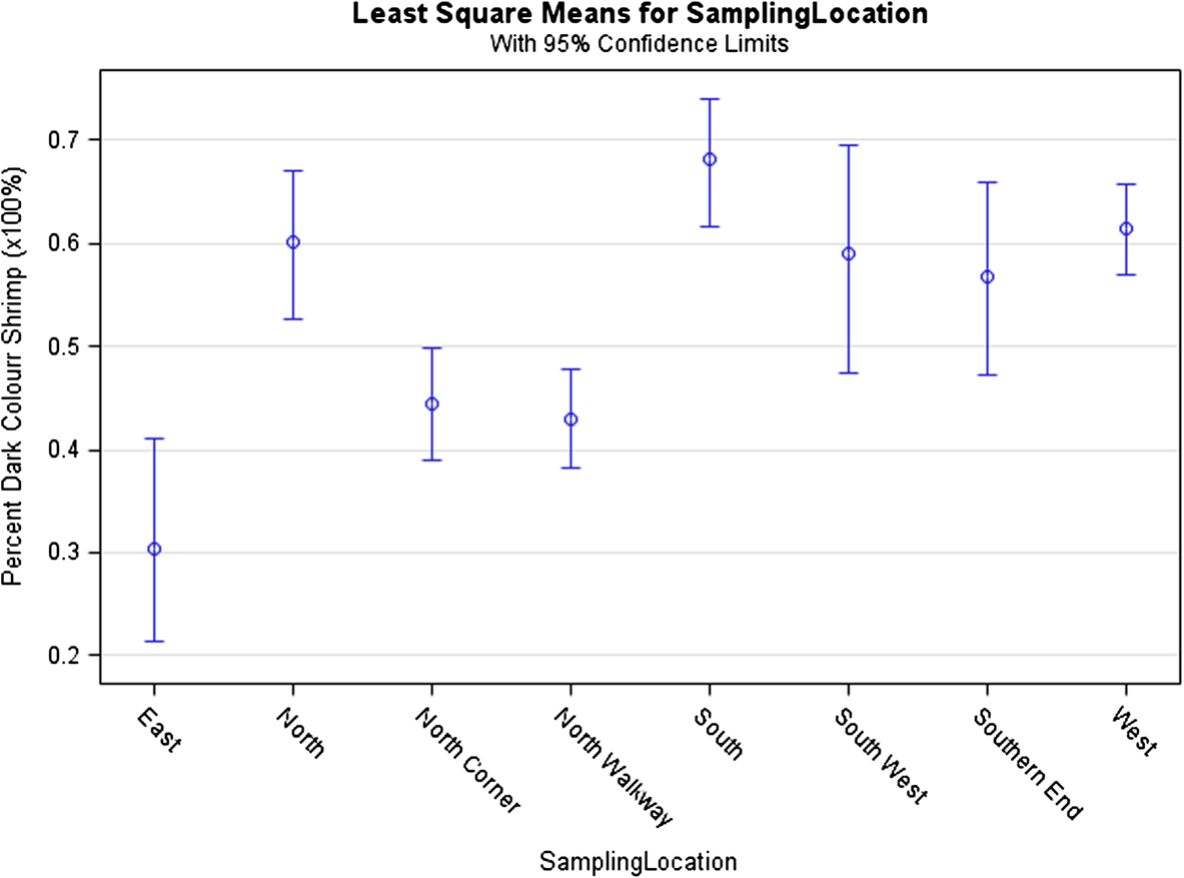
**Percentage of dark colour shrimp by sampling location.**

**Figure 3:**
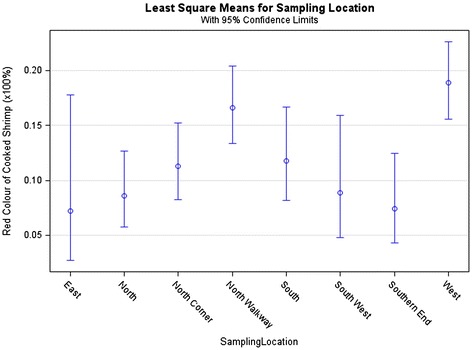
**Percentage of red colour of cooked shrimp by sampling location.**

The effect of sampling location in the site locations around the same pond was highly significant (P < 0.001) for all traits studied, including body colour of both raw and cooked shrimp (Figures 2 and 3). This was in part due to the very large size of grow-out pond (over 1 ha), hence environmental differences between sampling locations were possible even likely, and shrimp may have schooled according to size.

Banana shrimp females were substantially larger (P < 0.001) and heavier than males (Table 2). Conversely, the meat yield proportion of males was about 2.2% greater than that of females and the difference was statistically significant (P < 0.001). Between sex difference was observed for body colours of both raw and cooked shrimp (P < 0.05 to 0.001), that is, females had darker colour and a greater proportion of red coloured animals than males (Table 3).

**Table 2:** **Least squares means (± s.e.) for body and carcass traits by sampling time, operator and sex**

**Effects**	**Factors**	**WT**	**LG**	**HL**	**WD**	**TW**	**MY**
Sampling time	AM	17.6 ± 0.65	14.3 ± 0.19	5.0 ± 0.08	13.4 ± 0.28	10.6 ± 0.40	60.2 ± 0.42
	PM	15.9 ± 0.39	13.7 ± 0.12	4.8 ± 0.05	12.3 ± 0.15	9.6 ± 0.24	60.7 ± 0.25
Operator	1	16.3 ± 0.20	13.9 ± 0.06	5.0 ± 0.02	12.6 ± 0.07	10.2 ± 0.12	62.8 ± 0.12
	2	17.2 ± 0.21	14.1 ± 0.06	4.8 ± 0.03	13.1 ± 0.08	9.9 ± 0.13	58.1 ± 0.13
Sex	Female	17.8 ± 0.20	14.2 ± 0.58	5.0 ± 0.03	13.2 ± 0.07	10.7 ± 0.12	59.8 ± 0.13
	Male	15.6 ± 0.19	13.8 ± 0.06	4.8 ± 0.03	12.5 ± 0.07	9.5 ± 0.12	61.1 ± 0.13

WT = Live body weight, LG = Total length, HL = Head length, WD = Abdominal width, TW = Tail weight, MY = Meat yield = 100 × (Tail weight/ Body weight).

**Table 3:** **Least squares means (LSM ± s.e.), odds ratio (confidence interval, CI) for risk factors involved in body colours, ‘flesh streaks’ and yellow hepatopancreas**

**Effects**	**Levels**	**RC**		**CC**		**FS**		**YH**	
		**LSM**	**Odds ratio (CI)**	**LSM**	**Odds ratio (CI)**	**LSM**	**Odds ratio (CI)**	**LSM**	**Odds ratio (CI)**
Sampling time	AM	59.0 ± 7.39	1^f^	16.3 ± 6.31	1^f^	24.2 ± 0.09	1^f^	1.08 ± 0.01	1^f^
	PM	47.6 ± 4.58	1.57 (1.26 – 1.95)	10.3 ± 2.61	2.75 (2.03 – 3.71)	11.7 ± 0.03	2.43 (0.52-11.3)	0.005 ± 0.97	0.27 (0.12-.64)
Operator	1	44.2 ± 2.23	1^f^	11.6 ± 1.46	1^f^	16.4 ± 0.02	1^f^	0.02 ± 0.85	1^f^
	2	62.2 ± 2.28	0.49 (0.40 – 0.58)	14.6 ± 1.81	1.27 (0.99 – 1.62)	17.7 ± 0.02	0.91 (0.72–1.16)	0.10 ± 5.10	0.20 (0.08-.52)
Sex	Female	56.1 ± 2.32	1^f^	16.6 ± 1.92	1^f^	18.7 ± 0.02	1^f^	0.001 ± 0.15	1^f^
	Male	50.6 ± 2.34	1.22(1.01 – 1.46)	10.1 ± 1.37	0.54 (0.42 – 0.69)	15.5 ± 0.02	1.25 (0.98-1.60)	0.008 ± 0.09	0.61 (.30-1.26)

RC = Body colour of raw shrimp, CC = body colour of cooked shrimp, FS = ‘flesh streaks’ and YH = Yellow hepatopancreas.

^f^Reference.

The odds ratio (OR) coupled with its confidence interval obtained from generalised linear mixed model analysis also indicated that the proportion of red colour in cooked shrimp males was 46% less than in females (OR = 0.54, P < 0.01). However, the incidence of ’flesh streaks’ and yellow hepatopancreas shrimp was not different between the two sexes (P > 0.05) (Additional file 2 and Table 3).

### Heritability

The heritabilities for weight, length, width and tail (abdominal) weight were generally moderate to high, ranging from 14 to 50% (Table 4). Body colour of uncooked shrimp was moderately to highly heritable, but that of cooked shrimp tended to be lower (0.03 – 0.18). However, all the estimates had low standard errors and significantly different from zero (P < 0.05 to 0.01, z-score = 2.7 to 4.2).

**Table 4:** **Heritability (± s.e.) for traits of commercial importance in banana shrimp**

**Traits**	**Model**	**h** ^**2**^	**h** ^**2**^ **back-transformed**
Body weight	1	0.50 ± 0.08	
Body length	1	0.46 ± 0.07	
Head length	1	0.14 ± 0.03	
Width	1	0.45 ± 0.03	
Tail weight	1	0.49 ± 0.08	
Meat Yield	1	0.04 ± 0.02	
Colour of raw shrimp	1	0.18 ± 0.05	
	1	0.11 ± 0.03^A^	
	2	0.46 ± 0.11	0.33 ± 0.02
	2	0.29 ± 0.09^A^	0.21 ± 0.03
	3	0.55 ± 0.13	0.40 ± 0.02
	3	0.34 ± 0.11^A^	0.24 ± 0.03
Colour of cooked shrimp	1	0.08 ± 0.03	
	1	0.03 ± 0.02^A^	
	2	0.40 ± 0.13	0.18 ± 0.01
	2	0.18 ± 0.09^A^	0.08 ± 0.02
	3	0.41 ± 0.13	0.18 ± 0.01
	3	0.18 ± 0.09^A^	0.08 ± 0.02
‘flesh streaks’	1	0.03 ± 0.02	
	2	0.11 ± 0.06	0.05 ± 0.02
	3	0.11 ± 0.06	0.05 ± 0.02
Yellow hepatopancreas	1	0.02 ± 0.01	
	2	0.60 ± 0.36	0.04 ± 0.002
	3	0.35 ± 0.24	0.03 ± 0.002

Model 1 = Linear animal mixed model, Model 2 = threshold logistic model, and Model 3 = threshold probit model. ^A^Weight fitted as a linear covariate in the model.

Heritabilities (h^2^) for body colour were estimated using three different statistical models (Table 4). The linear animal mixed model (LMM) h^2^ (model 1) were low but significantly different from zero (ranging from 0.03 to 0.18). For linear sire model, the heritability for body colour of uncooked and cooked shrimp was 0.29 ± 0.08 and 0.12 ± 0.05, respectively (results not presented).

The generalised linear mixed model (GLMM) estimates of heritability for liability to body colour measurements used logit and probit models. The h^2^ obtained from logit model (model 2) were smaller than those from probit model (model 3) for raw colour. However, note that they cannot be directly compared because the estimates of heritability from the logit model were on the logistic scale whereas the ones obtained from the probit model were on the underlying normal scale. As expected from the theory, the GLMM estimates of heritability for body colour of raw and cooked shrimp on the original liability scale were markedly higher than those from LMM (ranging from 0.18 to 0.55 vs. 0.02 to 0.18, respectively). When the estimates on logit and probit liability scales were transformed to observable scale, heritabilities were quite similar between the LMM and GLMM methods, and statistically significant (P = 0.04 to <0.001, two tailed z-score = 3.8 to 15.1).

As expected, the hertiabilities for all morphometric traits were large, except for the meat yield which was not significant (P > 0.05).

### Correlations among morphometric traits and colour

The genetic correlations among body traits were all very high (Table 5). The near unity genetic correlations between body traits suggest that they are essentially controlled by the same set of genes and hence can be improved simultaneously in a selection program. All phenotypic correlations among body traits were consistent with genetic correlations and they ranged from 0.49 to 0.99.

**Table 5:** **Phenotypic (above) and genetic (below the diagonal) correlations among body and carcass measurements**

**Traits**	**WT**	**LG**	**HL**	**WD**	**TW**	**MY**
WT		0.99 (0.01)	0.49 (0.02)	0.93 (0.01)	0.98 (0.01)	−0.06 (0.03)
LG	0.99 (0.01)		0.51 (0.02)	0.88 (0.01)	0.92 (0.01)	0.02 (0.03)
HL	0.98 (0.04)	0.85 (0.08)		0.96 (0.04)	0.51 (0.02)	0.02 (0.03)
WD	0.99 (0.01)	0.99 (0.01)	0.95 (0.04)		0.93 (0.01)	0.01 (0.03)
TW	0.99 (0.01)	0.99 (0.01)	0.99 (0.03)	0.99 (0.01)		0.13 (0.03)
MY	−0.19 (0.22)	−0.27 (0.21)	−0.09 (0.25)	−0.19 (0.22)	−0.13 (0.22)	

Trait abbreviations given in Table 2.

All growth related traits showed positive genetic correlations with body colour of both cooked (0.74 to 0.84) and uncooked shrimp (0.59 to 0.70) (Table 6). The phenotypic correlations between body traits and colour were generally consistent in sign with those obtained for the genetic correlations, but they had significantly lower magnitude. The standard errors of both the phenotypic and genetic correlations were small but all the estimates were statistically significant (Table 6).

**Table 6:** **Phenotypic (r**_**P**_ **) and genetic (r**_**G**_ **) correlations of body and carcass traits with colour and yellow hepatopancreas and ‘flesh streaks’**

**Traits**	**RC**		**CC**		**YH**		**FS**	
	**r**_**G**_	**r**_**P**_	**r**_**G**_	**r**_**P**_	**r**_**G**_	**r**_**P**_	**r**_**G**_	**r**_**P**_
WT	0.66 (0.11)	0.28 (0.03)	0.84 (0.10)	0.20 (0.03)	0.85 (0.44)	0.13 (0.02)	−0.50 (0.30)	−0.11 (0.02)
LG	0.67 (0.11)	0.28 (0.03)	0.78 (0.12)	0.18 (0.03)	0.76 (0.64)	0.09 (0.02)	−0.98 (0.12)	−0.13 (0.03)
HL	0.59 (0.15)	0.16 (0.03)	0.74 (0.15)	0.08 (0.02)	0.59 (0.87)	0.04 (0.02)	−0.98 (0.12)	−0.13 (0.03)
WD	0.70 (0.11)	0.27 (0.03)	0.85 (0.11)	0.19 (0.03)	0.90 (0.34)	0.14 (0.02)	−0.60 (0.31)	−0.11 (0.02)
TW	0.61 (0.12)	0.27 (0.03)	0.84 (0.10)	0.20 (0.03)	0.99 (0.13)	0.18 (0.03)	−0.53 (0.30)	−0.11 (0.02)
MY	−0.54 (0.20)	−0.01 (0.03)	−0.05 (0.28)	−0.01 (0.02)	0.11 (0.48)	0.04 (0.02)	−0.12 (0.38)	0.05 (0.02)

Trait abbreviations given in Tables 2 and 3.

Standard errors in parentheses.

### Correlations among different measures of body colour

The genetic correlations between body colour of raw and cooked shrimp are high and positive (0.64 ± 0.20) (Table 7), indicating that this trait (red colour) is likely determined by the similar set of genes that give different phenotypes when measured in different environment (i.e. cooked vs. uncooked conditions). Interestingly body colour of cooked shrimp also showed a negative genetic correlation with ‘flesh streaks’ (−0.41 ± 0.36, P > 0.05), as expected from our visual observation. The genetic correlations between body colour measurements and yellow hepatopancreas were associated with large standard errors and not significantly different from zero (P > 0.05). All the phenotypic correlations were consistent in sign but they were of smaller magnitude compared with those obtained for the genetic correlations.

**Table 7:** **Phenotypic (above) and genetic (below the diagonal) between raw and cooked colour as well as with yellow hepatopancreas and ‘flesh streaks’**

**Traits**	**RC**	**CC**	**FS**	**YH**
RC		−0.22 ± 0.02	0.03 ± 0.02^ns^	−0.04 ± 0.02^ns^
CC	0.64 ± 0.07		−0.03 ± 0.02^ns^	−0.01 ± 0.02^ns^
FS	−0.35 ± 0.32^ns^	−0.41 ± 0.36^ns^		−0.05 ± 0.02
YH	0.74 ± 0.86^ns^	−0.43 ± 0.61^ns^	−0.67 ± 0.47^ns^	

^ns^ = non-significance.

Trait abbreviation given in Table 3.

## Discussion

The central objective of the present study was to understand if body colour of banana shrimp can be improved by genetic selection. The estimates of heritability achieved here suggest genetic improvement (by selective breeding) is possible for body colour, a trait of commercial importance in crustacean species, especially white leg shrimp *Litopenaeus vannamei* that accounts for a very large proportion (about 70%) of total crustacean production in Asia and Latin America. By using the genetic parameters estimates given in Tables 2 and 4, the predicted response to direct selection for red colour of cooked shrimp would be 8% per generation. Although body and carcass traits, meat yield as well as the potentially pathogen related traits of ’flesh streaks’ and yellow hepatopancreas were also examined, our discussion below placed emphasis on genetic basis of body colour and potential for future genetic improvement programs of this novel trait in banana shrimp and crustacean species.

### Heritabilities

This is the first study reporting genetic parameters for banana shrimp (*F. merguiensis*) and the first report of heritabilities for colour in crustacean. We have found there are large additive genetic variation observed for body colour (h^2^ = 0.03 – 0.55) and growth traits (h^2^ = 0.14 – 0.50), suggesting there is very good potential for genetic improvement of the traits studied in this population. The greater heritability for body colour in uncooked than cooked shrimp is an important finding since this shows that selection for shrimp colour can be practised on live breeding candidates. The improvement of cooked shrimp colour seems to be difficult since this character had a low heritability, perhaps due to large effects of environmental factors during cooking and storage as well as measurement methods. However selection for improving dark colour on live shrimp can improve redness of cooked animals as indicated by the high and positive genetic correlation between the two traits (see discussion on “correlations” section). Unfortunately, there are no prior genetic parameters reported for colour in crustaceans to compare with the estimates of this current study. In fish, heritabilities for flesh colour have been reported ranging from 0.09 to 0.32 [22-26]. Our results indicate the improvement of shrimp colour through direct selection or including colour with other traits in breeding objectives is practically feasible. Alternatively shrimp colour can be assessed perhaps more objectively and accurately, certainly more quantitatively using specialised instruments. In Australia the existing pricing systems reward producers for shrimp having higher colour scores than light-coloured counterparts. This would give an incentive to incorporate shrimp colour into practical genetic improvement programs. A rough calculation of economic benefit from one unit of improvement in body colour is about AU$ 2.6 million for the national sector ($2 increase per unit of improvement in colour × 1,300,000 kg = $2,600,000).

In addition to body colour, the large genetic variation in morphometric traits for banana shrimp in our study is consistent with those reported for other crustaceans species, such as pacific white shrimp (*P. vannamei*) [27,28], black tiger shrimp (*P. monodon*) [29], kuruma shrimp (*P. japonicus*) [30], redclaw crayfish (*C. quadricarinatus*) [31,32], and freshwater shrimp (*Macrobrachium rosenbergii*) [33,34]. The estimates of heritability for body traits in other species range from 0.20 to 0.60 [23,35-37]. Furthermore, ’flesh streaks’ and yellow hepatopancreas also showed significant genetic components (P < 0.05, z-score = 2.09), indicating that improvement for these characters can be achieved through conventional selection to improve flesh quality (i.e. reducing mushy, soft and chalky white texture) and disease resistance against possible pathogens.

In the present study, animals were pooled soon after hatching; hence, the maternal and common environmental effects (c^2^) were not significant as tested using the logarithmic likelihood ratio. However, in pedigreed populations where the c^2^ effects are present, they should be included in analytical model to avoid possible bias in genetic parameter estimates.

### Correlations

All quantitative traits measured in the current study including body traits and colour were genetically correlated. Among body traits, we found positive and high (almost unity) genetic correlations which agrees well with findings in Pacific white shrimp [38], freshwater shrimp *M. rosenbergii* [33], salmonids [39], and tilapia [40]. This suggests that all of the above body traits were closely genetically correlated and are likely to be influenced by similar sets of genes. The estimates of the genetic correlations here also suggest that any one of these traits tested could be used, on its own or simultaneously, to improve overall growth performance of the animals without a requirement for taking different measurements. However, in practical selection programs live weight or body length is recommended due to its greater heritability and the ease of measurements relative to other body dimensions (e.g. body width or carapace length).

The genetic correlations obtained in the present study between morphometric traits and body colour also allow the prediction of possible correlated changes when selection is practised on one trait or another. Due to the high and positive genetic correlations between weight and colour of raw (uncooked) shrimp, it is predicted that selection for increased harvest weight may result in favourable changes in colour of the shrimp and vice versa, that is, the animals selected for size become darker prior to cooking, or animals selected for darkness become heavier. Similarly, selection for higher weight would be accompanied by favourable increase in red colour of cooked shrimp. This is desired since red colour is a commercially important trait for the marketing of shrimp. Our results suggest that raw and cooked colours are under control of similar sets of genes but the genotype by environment interaction may be important as indicated by the significantly different from one genetic correlation between the two traits. They thus may be considered as genetically different traits in breeding programs. Comparison of our correlation estimates to other crustacean species is not possible due to unavailability of this information in the literature. However, in fish positive correlation between flesh colour and body traits have been reported for salmon [23,25,41] and tilapia [42]. The consistent results between body colour of shrimp and flesh colour in fish is likely that similar biological and metabolic pathways are involved in the process of controlling colour expressions in fish muscle and in exoskeleton (or hypodermal) tissues of shrimp. Genetic control of shrimp colouration is generally not well documented. Our estimates of the genetic correlations between growth related traits and shrimp colour are the first to indicate that indirect improvement in redness colour of cooked shrimp may be achieved from selection programs for high growth. It is however also necessary to develop alternative selection strategies to achieve optimal improvement in both performance and shrimp colour in the breeding programs.

### Environmental effects on body colour and performance of banana shrimp

Besides the significant genetic effects observed, environmental factors are well known to influence animal phenotypes, especially quality traits of economic importance in farmed aquaculture species [43]. In the present study, we found the sampling batch by location around the pond and sampling time had significant impacts on body colour of both cooked and uncooked shrimp, suggesting that without measuring such effects, sampling from ponds, at least large ones, could generate various biases in genetic parameter estimates. Female banana shrimp also had greater proportion of red colour after cooking than that evident in males. In fish, between- sex differences in flesh colour were thought likely due to sexual maturation effects [44] or due to the different degree of gonad development [45]. Besides the sampling batch by location and sex effects, other environmental factors have been reported to contribute to variation in body colour of shrimp, including background substrate colour of rearing environments, photoperiod, light intensity and temperature [3,46], moulting [47], storage, chilling, freezing and thawing process [6].

Furthermore, sex difference in growth traits was also observed in banana shrimp where females were about 21% heavier, on average, than males. This is also observed in *P. vannamei* where the divergence occurs at body weights of 10 to 17 g and females are significantly larger than males for most body traits including body weight (4.8%) and total length (1.2%) [48,49]. Sex dimorphism in growth and carcass yield have also been reported in many aquaculture species such as giant freshwater shrimp *M. roseinbergii* [50], tilapia *O. niloticus* [21], common carp [18], rainbow trout [51] or Atlantic salmon [39].

The significant effects of environmental factors on both shrimp colour and body traits shown in the present study suggest that all significant systematic effects, even the location of sampling in the pond, should be included in statistical models to analyse quantitative traits in genetic evaluation programs.

In summary, our study demonstrated there is a genetic (heritable) component for body colouration in banana shrimp, and hence there is potential for the improvement of this trait by genetic means. The application of DNA markers for parentage assignment can increase efficiency of the breeding program for this species by permitting communal rearing at a young age. With 10 ‘high quality’ microsatellite markers, 97.5% progenies were successfully assigned to single parental pair in our present study at high confidence. Both experimental and theoretical results show that by using 6–14 microsatellite markers, progenies can be assigned to the parents with a high degree of accuracy (90 to 99%) across aquatic animal species [52]. The DNA technology for genetic tagging has increasingly been applied in practical selective breeding programs [19,53-55]. Parentage testing and pedigree verification using DNA markers enables the conduct of genetic improvement programs under commercial production environments, without the need and cost of dedicated facilities and dedicated single pair mating design. This was demonstrated in our present study for *F. merguiensis.*

## Conclusions

In this study we indicated that microsatellite markers were successfully developed for *F. merguiensis* and these highly accurate pedigree assignment with high quality makers [9] was effective in permitting the use of commercial production ponds and samples for the estimation of genetic parameters. The mixed model estimates of genetic parameters in the present study indicate that body colour of the shrimp can respond effectively to selection. Selection for dark colour on live shrimp is also expected to increase redness of cooked animal. The genetic association of body colour of raw and cooked shrimp with morphometric traits were high and positive, suggesting that both body colour and morphometric traits can be easily improved simultaneously in breeding programs for this species. Genetic improvement of body colour in crustaceans is foreseen as a sustainable alternative to the addition of feed additives to animal diets due to consumers’ concerns regarding food safety issues and there has been a growing public interest in environmentally friendly products. The improvement of colour by genetic means is expected to bring about potential economic benefits to the shrimp sector world-wide.

## Additional files

Additional file 1
**Characteristics of the 10 polymorphic microsatellites tested on 48 wild caught** ***Fenneropenaeus merguiensis*** **individuals:** ***A*** **, number of alleles per locus;** ***PIC*** **, Polymorphic Information Content;** ***H***_***O***_ **, observed heterozygosity;** ***H***_***E***_ **, expected heterozygosity under conditions of Hardy-Weinberg equilibrium.**
Additional file 2
**Analysis of variance for body and carcass traits, shrimp colour and hepatopancreas.**
