# Supplementary material for: Heritability for body colour and its genetic association with morphometric traits in Banana shrimp (Fenneropenaeus merguiensis)
Source: BMC Genet. 2014 Dec 5;15:132. doi: 10.1186/s12863-014-0132-5 (PMC4261751; doi:10.1186/s12863-014-0132-5)
Supplement: Additional file 1 — Characteristics of the 10 polymorphic microsatellites tested on 48 wild caught Fenneropenaeus merguiensis individuals: A , number of alleles per locus; PIC , Polymorphic Information Content; H O , observed heterozygosity; H E , expected heterozygosity under conditions of Hardy-Weinberg equilibrium. [file 12863_2014_132_MOESM1_ESM.docx]

Additional file 1: Characteristics of the 10 polymorphic microsatellites tested on 48 wild caught *Fenneropenaeus merguiensis* individuals: *A*, number of alleles per locus; *PIC*, Polymorphic Information Content; *H_O_*, observed heterozygosity; *H_E_*, expected heterozygosity under conditions of Hardy-Weinberg equilibrium.

| **Locus and GenBank Accession**  **Number** | **FWD Primer** | **REV Primer** | **Repeat motif** | **Size range**  **(bp)** | ***A*** | ***PIC*** | ***H_O_*** | ***H_E_*** | **Dye**  **Label** |
| --- | --- | --- | --- | --- | --- | --- | --- | --- | --- |
| FM001  KM213743 | GCGATGACAATAATAACACTATCAA | AGGTAATGATGACGGTTCAAGA | (TAA)_23_ | 107-155 | 17 | 0.910 | 0.938 | 0.925 | FAM |
| FM002  KM213744 | CCAAAACAACTAAACCCAAACA | GGGGTCTTACTTAGTCTTTGATCG | (CAT)_16_ | 160-217 | 22 | 0.863 | 0.660 | 0.883 | FAM |
| FM004  KM213745 | ACTGGGTAAGGCGATGAATG | TGAACCAATACAAGCTCCAGTG | (CAA)_19_ | 111-131 | 8 | 0.731 | 0.682 | 0.772 | NED |
| FM005  KM213746 | TGACGTTAATAATGGGAAGATGG | CCCTTGCACTTTCTATTCGAT | (ATG)_30_ | 222-326 | 22 | 0.871 | 0.804 | 0.890 | NED |
| FM011  KM213747 | TCCGTTGTGAGACCGATACA | GGGAAAAGGAAATTTCTGTGG | (TG)_59_ | 213-283 | 10 | 0.366 | 0.296 | 0.381 | PET |
| FM014  KM213748 | TCCGCGTGTTTACCAATACA | CCTTCACTCTCCCTACAACACC | (GGGA)_28_ | 234-261 | 9 | 0.607 | 0.563 | 0.648 | PET |
| FM047  KM213749 | AGAAAGGAACGAAAAGGGGA | TCTCATTATGACTATTAGCATTAGCGT | (ATG)_9_ | 114-151 | 11 | 0.589 | 0.463 | 0.613 | FAM |
| FM052  KM213750 | GTGGATTCCCTCATGTGGAC | CTTCCCCTTTTCATTCCCTC | (AGGG)_8_ | 132-158 | 4 | 0.565 | 0.442 | 0.621 | NED |
| FM056  KM213751 | TGTTTGTCCTATGGGGTATGC | CCTGATAAGCTGTGGAGACGA | (GCGTGT)_8_ | 187-236 | 8 | 0.706 | 0.742 | 0.752 | FAM |
| FM057  KM213752 | AGCACCAGGAGGAAGAGGAG | GAACGCGATCTGTGTTGAGA | (GAA)_8_ | 139-165 | 8 | 0.452 | 0.521 | 0.494 | PET |
