# Supplementary material for: Heritability for body colour and its genetic association with morphometric traits in Banana shrimp (Fenneropenaeus merguiensis)
Source: BMC Genet. 2014 Dec 5;15:132. doi: 10.1186/s12863-014-0132-5 (PMC4261751; doi:10.1186/s12863-014-0132-5)
Supplement: Additional file 2 — Analysis of variance for body and carcass traits, shrimp colour and hepatopancreas. [file 12863_2014_132_MOESM2_ESM.docx]

Additional file 2: Analysis of variance for body and carcass traits, shrimp colour and hepatopancreas

| Effects | DF | Body and carcass traits | | | | | |  | Body colours and diseases | | | |
| --- | --- | --- | --- | --- | --- | --- | --- | --- | --- | --- | --- | --- |
|  |  | WT | LG | HL | WD | TW | MY |  | RC | CC | FS | YH |
| Sampling location*batch | 24 | 6.5^***^ | 5.7^***^ | 6.3 ^***^ | 5.7^***^ | 6.3^***^ | 2.2^***^ |  | 10.3^***^ | 4.9^***^ | 2.5^**^ | 1.6 ^ns^ |
| Time (AM or PM) | 1 | 2.9 ^ns^ | 3.6 ^ns^ | 2.8 ^ns^ | 6.7 ^**^ | 2.2 ^ns^ | 0.7 ^ns^ |  | 16.6^***^ | 43.2^***^ | 18.0^***^ | 9.0 ^**^ |
| Operator | 1 | 20.1^***^ | 6.8^***^ | 53.9^***^ | 58.6^***^ | 3.5 ^ns^ | 1332.3^***^ |  | 59.2^***^ | 4.2^ns^ | 0.4 ^ns^ | 10.9^**^ |
| Sex | 1 | 110.7^***^ | 49.4^***^ | 65.2^***^ | 109.9^***^ | 81.3^***^ | 95.2^***^ |  | 4.4^*^ | 23.1^***^ | 3.7 ^ms^ | 1.51 ^ns^ |

^*^P < 0.05, ^**^P <0.01 and ^***^P <0.001, ns = non=significance, ms = marginal significance (P = 0.056) and DF = Degree of freedom

Trait abbreviations given in Tables 2 and 3
